# Biplanar Nulling Coil System for OPM-MEG Using Printed Circuit Boards

**DOI:** 10.3390/s25092759

**Published:** 2025-04-27

**Authors:** Mainak Jas, John Kamataris, Teppei Matsubara, Chunling Dong, Gabriel Motta, Abbas Sohrabpour, Seppo P. Ahlfors, Matti Hämäläinen, Yoshio Okada, Padmavathi Sundaram

**Affiliations:** 1 Athinoula A. Martinos Center for Biomedical Imaging, Department of Radiology, Massachusetts General Hospital, Boston, MA 02129, USAtmatsubara@mgh.harvard.edu (T.M.); padma@nmr.mgh.harvard.edu (P.S.); 2 Harvard Medical School, Boston, MA 02115, USA; 3 Department of Neuroscience and Biomedical Engineering, School of Science, Aalto University, 02150 Espoo, Finland; matti.s.hamalainen@aalto.fi; 4 Boston Children’s Hospital, Boston, MA 02115, USA

**Keywords:** magnetoencephalography, field nulling, optically pumped magnetometer, printed circuit boards, somatosensory evoked field

## Abstract

Optically pumped magnetometers (OPMs) are a promising magnetoencephalography (MEG) technology for the non-invasive measurement of human electrophysiological signals. Prior work developed biplanar background field-nulling coils necessary for OPM operation, but these were expensive to produce and required tedious error-prone manual winding of >1 km of copper wire. Here, we developed a precise and reproducible manufacturing process by fabricating these coils on two-layer printed circuit boards (PCBs). Building on open-source software (bfieldtools), we developed a pipeline to determine the optimal current loops of 1.5 × 1.5 m^2^ biplanar nulling coils, connected these loops into a continuous conducting path across PCB layers, and printed them as pairs of 1.5 × 0.75 m^2^ PCBs, which were soldered and mounted on an aluminum frame. Our coils achieved efficiencies of 1.3–7.1 nT/mA, similar to or higher than previous designs. We reduced the largest background field component from 21 to 2 nT, enabling OPMs in a lightly shielded room to record somatosensory evoked fields (SEFs) comparable to SQUID-MEG. Our coil system is cheaper than commercial alternatives and is available as an open-source package opmcoils, thus enabling more affordable background field nulling for OPM-MEG and realizing its potential as an accessible sensor technology for human neuroscience.

## 1. Introduction

Optically pumped magnetometers (OPMs) are a promising new sensor technology for magnetoencephalography (MEG). They allow for the measurement of weak magnetic fields from the brain at room temperature [1]. Unlike conventional cryogenic superconducting quantum interference device (SQUID) sensors, OPMs do not require a fixed gantry and can be placed in a flexible manner on the subject, thus expanding the potential applications of MEG. OPMs have been used, for instance, in naturalistic paradigms involving movements [2] as well as in pediatric MEG studies [3]. When placed on the scalp, OPM sensors may also improve the spatial resolution obtainable using MEG [4,5]. Despite the potential of OPMs, they have not been widely adopted [6], partly because they require a near-zero background field for optimal operation, which remains a challenge [7,8]. The near-zero background field is necessary to avoid saturation and non-linearity of the OPM sensors [9]. In addition to the cancellation of the uniform field, minimizing the field gradient is important for reducing artifacts due to movement [10].

On-sensor Helmholtz coils built into OPM sensors can null background fields also in closed-loop mode, enabling continuous nulling even when the background field drifts. Depending on the sensor design, the dynamic range varies from ±15 nT [7] to ±200 nT [11] in closed-loop mode. Any sensor movement that changes the background field greater than this dynamic range will cause the sensor to saturate or move outside the linear operating regime [9]. More importantly, due to cross-axis projection error (CAPE), even small uncorrected background fields that allow the sensor to operate may distort the measurements by introducing phase and amplitude errors along all axes, causing source localization errors of up to 1 cm [8]. Finally, as OPMs move towards lighter shielding in challenging clinical environments, large currents in on-sensor coils may magnify cross-talk effects. These considerations may assume greater importance as OPM-MEG studies start using more tightly packed sensors to fully leverage the spatial frequencies on the scalp surface [4,5,12].

On-sensor Helmholtz coils built into OPM sensors can null background fields also in closed-loop mode, enabling continuous nulling even when the background field drifts. Even then, the dynamic range of the sensors is limited to ±15 nT in closed-loop mode [7]. Any sensor movement that changes the background field greater than this dynamic range will cause the sensor to saturate or move outside the linear operating regime [9]. On-sensor coils may introduce cross-talk and may be challenging to operate in lightly shielded rooms [7,13].

**Figure 1:**
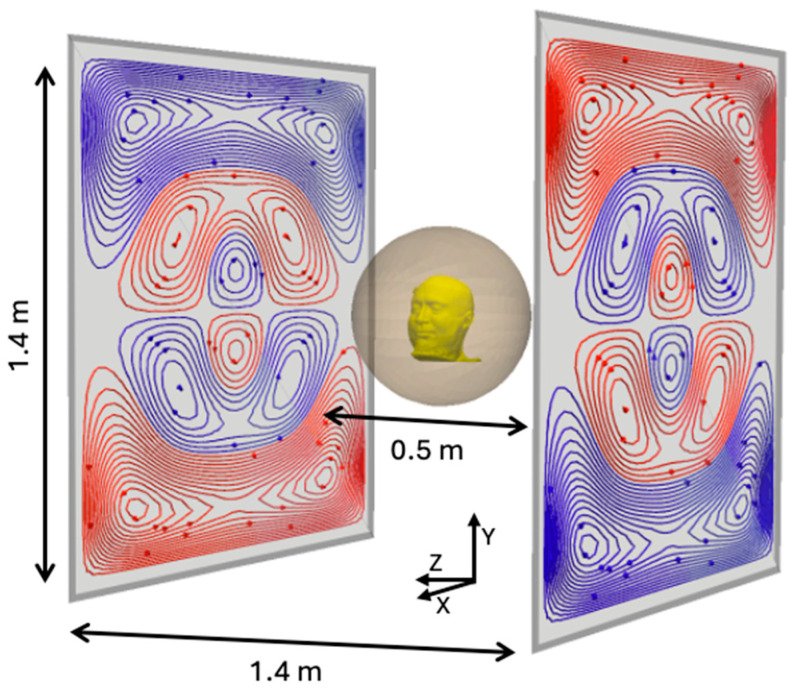
The overall dimensions of our field-nulling system. 1.4 × 1.4 m^2^ coils were etched in a PCB pair of size 1.5 × 1.5 m^2^ and separated by 1.4 m. The target region is a sphere with a diameter of 50 cm.

To address these problems, field-nulling coil systems have been developed to minimize the background field (uniform and gradients) inside a target region containing the subject’s head [14,15,16]. Simple Helmholtz coil-based designs [16] are efficient, but they are not always appropriate for neuroimaging studies as the coils may obstruct access to the subject. Biplanar “fingerprint” coils have been designed [1,14,15,17] where the current distribution is restricted to a planar surface. They allow field nulling inside a target region using mathematically optimized current distributions (Figure 1). Biplanar coils trade lower efficiency, i.e., magnetic field generated per unit current applied to the coils, for improved usability and access to the subject. Newer systems using matrix coil designs [18] are being developed, but they are less efficient, more complex to operate, and more expensive due to the number of independent current drivers required for their operation. Biplanar “fingerprint” coils therefore remain the most common choice for background field nulling in OPM systems.

A challenge with existing biplanar coil manufacturing [14,15] is that it depends on the manual winding of large quantities (>1000 m) of copper wire, a complex process that is inherently labor- and time-intensive. To construct such a coil, the winding pattern is printed on a sheet of paper, then wire is glued onto it using epoxy, and the assembly is supported using a medium-density fiberboard. To minimize the resistance and thermal noise, a low gauge (thick) wire is used, making winding difficult. Even though a skilled individual may be able to wind the coils in a short amount of time, it could pose significant challenges to scale and reproduce. To improve the ease of construction, enhance reproducibility, and avoid manual winding, our goal was to automate as much of this process as possible. Printed circuit boards (PCBs) present a promising solution by allowing high-precision etching of the desired current path. However, PCB-based coils are not straightforward to design with existing tools because they yield disjointed current loops which must be fully connected into a continuous conducting path that traverses the PCB layers.

Here, we present an open-source semi-automated pipeline for manufacturing nulling PCB coils that addresses the practical challenges with existing biplanar coil systems. PCBs allow for precision in manufacturing and easy replication since the design files can be shared. Our open-source package opmcoils, contains the Python-based software needed to design the nulling coils, the design files used to print our PCBs, and installation instructions. We found that the material cost of our system was significantly less than the cost of currently available commercial nulling coil systems. We expect this to contribute to more affordable and easily available field-nulling options for OPM-MEG.

In this paper, we outline our process and discuss the challenges we faced in using the newly developed tools. We characterized the accuracy and efficiency of the coil system and compared experimental data to theoretical values. We mapped the residual field before and after using the field-nulling system. We also demonstrate somatosensory evoked responses recorded with OPMs operating in the environment created by the PCB coils in a one-layer shielded room, providing data comparable to that measured by SQUIDs in a three-layer room.

## 2. Materials and Methods

We developed opmcoils, a software tool that provides an application programming interface (API) for coil design and abstracts away the technical implementation details. The purpose was to catalyze future development of the field-nulling systems by allowing developers to obtain the current loops, connect them into a continuous path, evaluate the coil efficiency as a function of design parameters (e.g., coil size, inter-coil distance, shielded room), and export the conducting paths to manufacturable PCBs. Below, we describe the fabrication, installation, and evaluation of the field-nulling PCB coils designed to be used for OPM-MEG in a lightly shielded room.

### 2.1. Pipeline for Manufacturing Biplanar Nulling PCB Coils

#### 2.1.1. Optimizing and Discretizing Stream Functions

The target field method has been widely used in MRI for gradient coil design [19,20,21,22] as well in transcranial magnetic stimulation [23,24] The underlying physical principles and the optimization problem specific to OPM-MEG nulling coil design are adequately addressed in the literature [14,15,25,26]. We used bfieldtools, a Python-based open-source software package for magnetostatic calculations on surfaces of arbitrary shape [25,26].

Bfieldtools represents magnetic fields using spherical harmonic functions and the unknown current densities using stream functions. The stream functions are restricted to a triangle mesh representing the coil surface. The estimation of the unknown stream function s is a convex optimization problem with a quadratic objective function,

$$\hat{s}=\underset{s}{\mathrm{argmin}}\frac{1}{2}s^{T}Rs+\lambda {\left|\left|b-As\right|\right|}^{2}\;$$

where R is the resistance matrix, A is the coupling matrix that maps the stream function s to the measured magnetic field b, and λ is the regularization parameter. One can think of the minimization of the quadratic term $s^{T}Rs$ as minimizing the resistive loss in the coil.

**Figure 2:**
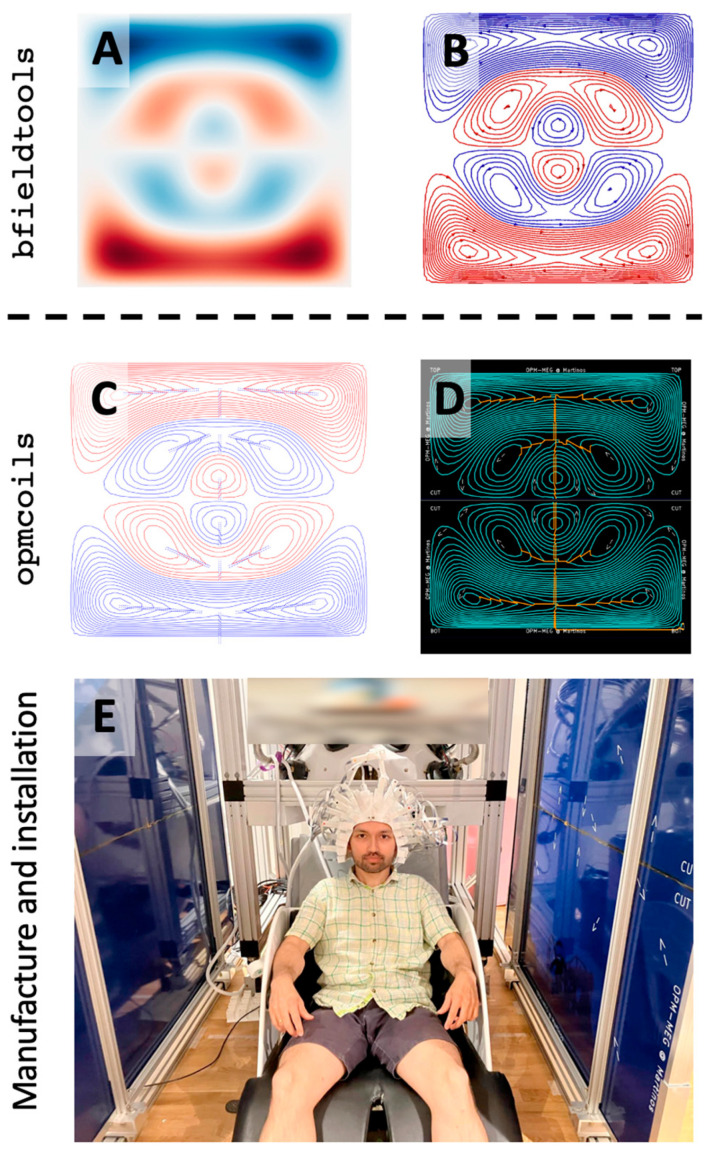
Pipeline for manufacturing biplanar nulling coil system using printed circuit boards. Existing open-source tools only allow for the optimization and discretization of the stream functions (**A**,**B**) but hardware realization of the optimized loops is expensive and non-trivial. We developed opmcoils (**C**,**D**) to enable the development, evaluation, and manufacture of biplanar nulling coils. The nulling coils can then be used in OPM-MEG experiments, as demonstrated here by the first author (**E**).

Under the hood, bfieldtools uses CVXPY [27] to solve the quadratic optimization problem. Once the estimate $\hat{s}$ was obtained from bfieldtools (e.g., Figure 2A), it was discretized to obtain a certain number of turns (we used N = 30, Figure 2B). These optimizations were carried out for each of the three uniform field coils ($B_{x}$, $B_{y}$, $B_{z}$), and for each of the gradient coils ($G_{xz}=\frac{\partial B_{x}}{\partial z}$, $G_{yz}=\frac{\partial B_{y}}{\partial z}$, $G_{zz}=\frac{\partial B_{z}}{\partial z}$). Since, in a current-free region, ∇×B=0, our coils can also null $G_{zx}$ and $G_{zy}$ [14]. The *z*-axis was defined to be normal to the coil planes, going through the center of the coils, *y*-axis vertical from floor to ceiling, and *x*-axis parallel to the coil planes (Figure 1).

We extended the capability of bfieldtools to compute the efficiency of the discretized coils with a given number of turns and trace width. Here, coil efficiency was defined as the magnitude of the magnetic field generated per unit of current applied to the coil (in units of nT/mA for the uniform field coils or nT/m/mA for the gradient coils). Once the efficiency of the designed coils was found to be sufficient, the remaining challenge was that the discretized current loops were disconnected from each other. In the next section, we describe how these loops were connected into a single continuous path traversing the two layers of the PCB while minimizing stray magnetic fields.

#### 2.1.2. Connecting Current Loops

**Figure 3:**
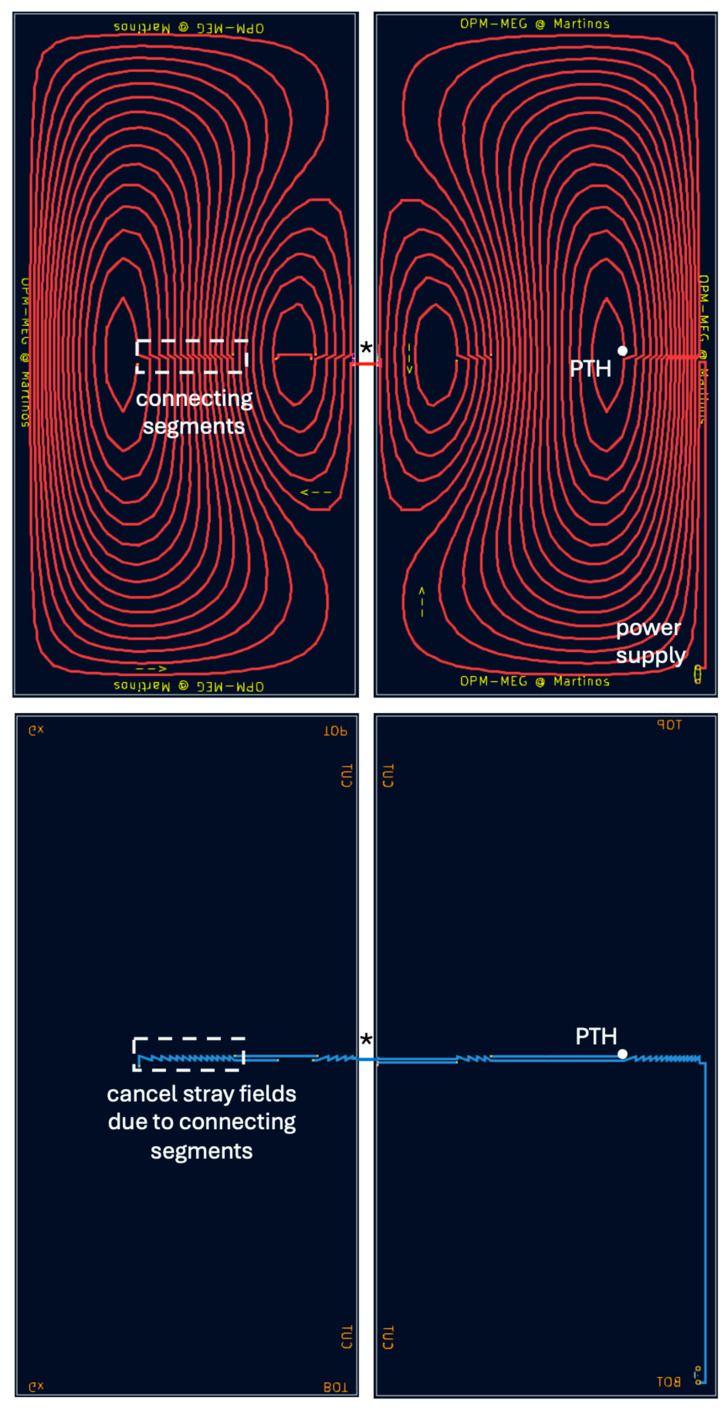
Completed two-layer nulling coil design (*G_xz_*) visualized in KiCad, the electronic design software. opmcoils generated the wire paths to connect the discretized loops into a continuous conducting path in each PCB. The connecting segments were identical in the front (**top**) and back layer (**bottom**) of the PCB but with opposing current directions, thus self-canceling any additional stray fields. Plated through holes (PTHs) connected the front and back layer at specific locations. The coil was cut along the symmetry axis into two PCBs (left and right columns). During installation, the front and back layers of the PCBs were soldered at designated solder masks (*) to create a complete conducting path.

**Figure 4:**
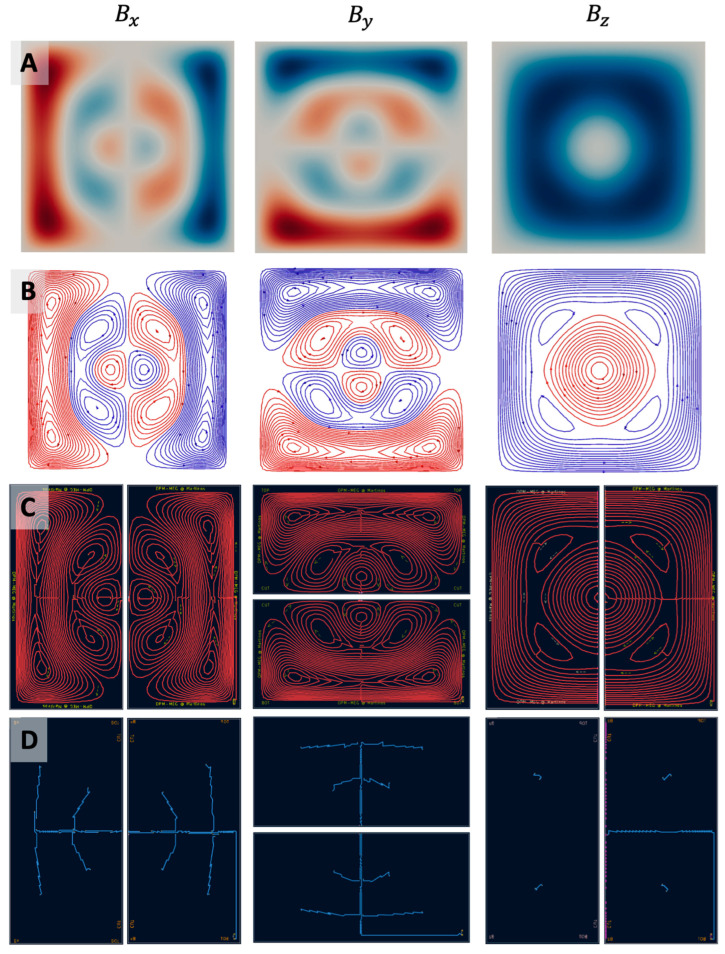
Completed designs for uniform field (*B_x_*, *B_y_*, and *B_z_*)-nulling coils starting from optimized stream functions (**A**), discretized current loops (**B**), and connected current paths in the two-layer PCBs ((**C**) front layer, and (**D**) back layer).

**Figure 5:**
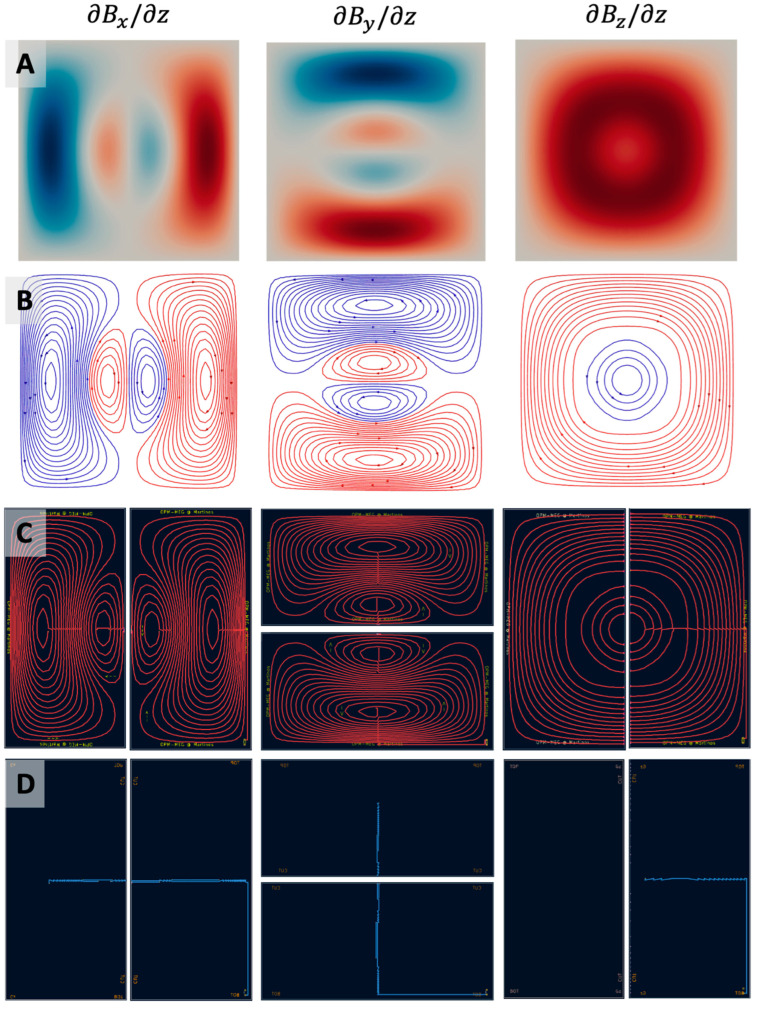
Completed designs for field gradient ($\partial B_{x}/\partial z$, $\partial B_{y}/\partial z,$ and $\partial B_{z}/\partial z$,)-nulling coils starting from optimized stream functions (**A**), discretized current loops (**B**), and connected current paths in the two-layer PCBs ((**C**) front layer, and (**D**) back layer).

We developed an interactive Python tool to join the discretized current loops into a continuous path. The discretized current loops needed to be either clockwise (Figure 2B: red) or anticlockwise (Figure 2B: blue). Neighboring loops with the same current direction were connected in series along a line segment drawn interactively with our software (Figure 2C). As the current path spiraled inward or outward, it reached the innermost or outermost loop. Since the additional wire segments used to connect the discrete loops together were not part of the optimized coil design, they were canceled with opposing current paths in the second layer (Figure 2D). At the end of this step, the connected current loops formed “islands” which were disconnected from each other and from the power terminals of the PCB. The final step was performed manually; we exported the connected current loops to KiCad (https://www.kicad.org/), a free open-source electronic design software for PCB manufacturing. We designed the coils for 1.5 × 1.5 m^2^ square PCBs; however, due to manufacturing size constraints, the coil needed to be printed in two 1.5 × 0.75 m^2^ pieces. Therefore, during export to KiCad, our Python code cut the connected coil traces along the axis of symmetry (vertical cuts for $B_{x}$, $G_{xz}$, $B_{z}$, $G_{zz}$; horizontal cuts for $B_{y}$, $G_{yz}$ coils; Figure 3). Cutting along the symmetry axis meant that at least for the $B_{x}$, $B_{y}$, $G_{xz}$, and $G_{yz}$ coils, only two solder joints (one for each layer) would be required to connect the two pieces (Figure 4C,D and Figure 5C,D). Only the $B_{z}$ and $G_{zz}$ coils needed multiple solder joints to connect the two pieces (Figure 4C,D and Figure 5C,D). Below, we explain the final step in KiCad to create printable design files for PCB manufacture.

#### 2.1.3. Printed Circuit Board Design

We used two-layer PCBs with standard 2 oz copper: each board contained a front copper layer and a back copper layer (Figure 3). Readers may note that the thickness of a PCB is typically specified in oz, which is a shorthand for oz/ft^2^. Thus, when the PCB thickness is specified as 1 oz, it refers to the thickness of the copper sheet when 1 oz copper is flattened on to a surface area of 1 ft^2^. In SI units, 1 oz Cu would be 35 microns thick. The front layer traced the main coil pattern, and the back layer was used to “lift the pen” and move from one set of the current loops to another, without crossing paths on the coil patterns in the plane. We set the PCB trace width to the maximum feasible (5 mm) to minimize the coil resistance. To connect one layer to another, we used 2 mm diameter copper plated through holes (PTHs). The current loop “islands” were connected to each other and to the power terminals on the PCB using the wire drawing tool in KiCad. Wire segments measuring 5 mm were drawn along the horizontal or vertical directions with the return paths in the back layer canceling any stray magnetic fields. During the KiCad finishing step, we annotated the coils (text labels indicating cut locations and current directions for the different loops). These annotations were made on the silkscreen layers (one for the front, one for the back) to help with the assembly after manufacture. At the cut edges on front and back, we added solder pads for soldering the boards together during installation. The power terminals were added and aligned across all the coils.

#### 2.1.4. Manufacturing and Installation

The final coil designs were exported to Gerber format files and sent for manufacturing (Shenzhen Hopetime Industry Co., Ltd., Shenzhen, China). Gerber files are a common PCB design format that stores the shape and location information for every element in the PCB. Each layer of the PCB (in our case, front and back Cu, front and back silkscreen for annotation and a drill file with the locations of the PTH) was exported into its own Gerber file. A key design consideration was the ability to test and replace coils if necessary. The coils were therefore designed to be stacked together with an 8 mm inter-coil spacing. We accounted for this spacing during the coil design. We first printed and tested the $B_{y}$ coil pair. Once we were satisfied with the performance of the first coil pair, the remaining coils were printed. For all the printed coils, the two PCB halves were manually soldered together. Since the PCBs were large and heavy, we designed a frame to mount the entire nulling coil system and hold it upright to prevent buckling. Our frame design allows the mounted PCBs to slide on rails depending on the study requirements.

Using the pipeline outlined above, we designed and fabricated six coils: three uniform field coils ($B_{x}$, $B_{y}$, $B_{z}$), and three gradient coils ($G_{xz}$, $G_{yz}$, $G_{zz}$). Since, in a current-free region, ∇×B=0, our coils can thus also null $G_{zx}$ and $G_{zy}$ [14].

### 2.2. Theoretical Estimation of Coil Resistance and Efficiency

We computed the theoretical resistance for the discrete current loops and the joined current path on the PCB for each coil. We used bfieldtools to compute the total length of the disconnected discretized current loops and opmcoils to compute the total length of the coil which will be printed into a continuous path on the PCB. The resistance of the coil is a function of the total length, the PCB trace width, and the density of the copper coating (2 oz Cu corresponding to 70 μm Cu thickness). Increasing the copper weighting on a large PCB can be quite expensive but one can easily increase the trace width to the widest feasible width (5 mm) without any increase in cost or difficulty in winding. Lower coil resistance reduces thermal noise from the current driver, thus improving the overall quality of the applied nulling field.

The theoretical efficiency (in units of nT/mA or nT/m/mA) of each of the six coils was calculated with and without accounting for the shielded room. We estimated the theoretical efficiency of the coils without the presence of the shielded room. In addition, we used the approach of [25,26] to model the effect of field distortions due to the high permeability mu metal in the floor, walls, and ceiling of our shielded room. We modeled the mu metal as an infinite permeability material that requires an additional boundary condition. The boundary condition was satisfied by setting the scalar magnetic potential at the inner shield to 0 and introducing an equivalent stream function for the magnetic shield:

$$C_{U_{coil}}s_{coil}=-C_{U_{shield}}s_{shield}$$

where $C_{U_{coil}}$ and $C_{U_{shield}}$ are the magnetic scalar potential coupling matrices. With this equipotential boundary condition, the total magnetic field at the target points was computed as:

$$B=C_{B_{coil}}s_{coil}+C_{B_{shield}}s_{shield}=\left(C_{B_{coil}}-C_{B_{shield}}C_{U_{shield}}^{-1}C_{U_{coil}}\right)s_{coil}$$

These values were used to calculate the theoretical estimate of the coil efficiency accounting for the effect of the shielded room. Both calculations were incorporated in opmtools.

### 2.3. Empirical Measurement of Coil Resistance and Efficiency

We measured the total resistance of each coil pair with a multimeter (Fluke 115 from Fluke, Everett, WA, USA). We used low-noise bipolar constant current drivers (CSB-40, CSB-100 from TwinLeaf, Princeton, NJ, USA) each with three independent outputs. The 100-mA current driver (CSB-100) was used for $B_{x}$ and $B_{y}$ coils since they were theoretically less efficient than the other coils. For each coil pair, we measured the background field at the center of the target region as a function of the applied current (0 to 60 mA). The background field was measured using a fluxgate magnetometer (FVM400, Meda, Dulles, VA, USA). The efficiency of the uniform field coils was computed as the slope of the best fitting line to the measurements. Similarly, to measure the gradient, we 3D-printed an array of sensor holders spaced 5 cm apart between −15 cm and 15 cm along the z-axis (x = 0, y = 0; middle of the nulling coils). The efficiency of the gradient field coils was computed by varying the measured gradient as a function of the applied current (0 to 24 mA).

### 2.4. Mapping the Remnant Field Inside the Target Area

**Figure 6:**
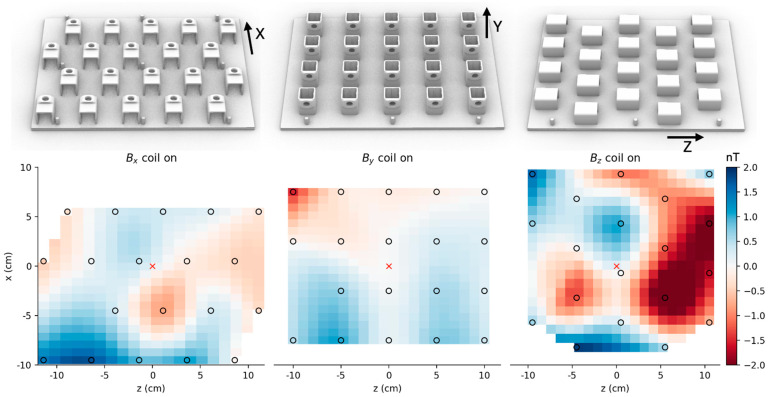
Spatial maps of the residual field when nulling coils corresponding to the x, y, and z directions are turned on one at a time. (**Top**) Model of the 3d-printed sensor holders used for mapping. A pegboard was used to align the maps across the three measurements. (**Bottom**) Spatial maps after turning on the $B_{x}$, $B_{y}$, and $B_{z}$ nulling coils, respectively. The maps were centered at the target region (red cross) in the x-z plane. The positions of the centers of the OPM’s vapor cells are shown in black circles. Field values elsewhere were interpolated.

We used OPMs to map the spatial profile of the residual magnetic field in the target zone in the x-z plane after the uniform field coils were applied, sampled at a 5 cm resolution in a 400 cm^2^ region (Figure 6, bottom). Sensor holders for measuring the x, y, and z components of the field were 3d-printed along with a reference pegboard to align the measurements (Figure 6, top). The measured magnetic field was the remnant field nulled by the on-sensor coils in the OPMs [7]. We used 19 single-axis OPMs (Fieldline Gen 2, Boulder, CO, USA) with their sensitive axes along the measured field component. The effect of time-varying drifts in the background field were minimized by measuring the remnant field simultaneously from all the OPM sensors. The bias in the remnant field estimate for each OPM sensor was measured by averaging two observations: one in the original position and another with the sensor flipped along its sensitive axis. The field maps were produced after correcting for this (measurement) bias.

### 2.5. Estimating Optimal Applied Current on a Helmet

An automated procedure was devised to set the currents to minimize the background field in the OPM sensors inside a helmet. Following the method of [16], we used a procedure that does not require any additional sensors to determine the applied current. The fields b in OPM sensors corresponding to the nulling coil currents I were represented using a coupling matrix M, such that b=MI. By driving the nulling coils one at a time with known currents, the columns of coupling matrix M can be experimentally determined. Then, to null a measured field b with known coupling matrix M, the driving currents can be estimated from $I=M^{-1}b$.

The automatic nulling procedure zeroed the fields at target sensors without explicitly estimating the uniform and gradient components of the background field. This method worked best when the coils produced dissimilar measurements so that the coupling matrix M had a reasonably low condition number [16]. Accordingly, we placed the sensors in a subject-specific helmet (while not being worn by the subject) where the sensors are oriented in multiple directions. The residual field on applying the optimal current was measured using OPMs and mapped on the helmet surface.

### 2.6. MEG Measurement and Analysis of Somatosensory Evoked Fields (SEF)

We performed a median-nerve stimulation experiment [28] on one healthy adult subject (male, 35 years old). The participant provided written informed consent. The study design, protocol, and consent form were approved by the Massachusetts General Hospital Institutional Review Board. We first performed the experiment using conventional SQUID-based MEG (306-channel Triux neo system, MEGIN Oy, Espoo, Finland). The subject was asked to stay awake and keep their head still for the duration of the experiment (4 min). The median nerve on the right wrist was stimulated with inter-stimulus interval of 500 ms. We repeated the experiment in OPM-MEG (19 magnetometers, FieldLine Gen 2, FieldLine, Boulder, CO, USA) using the same paradigm. The field-nulling coils were operated, and the currents were set using the approach in Section 2.5 (as demonstrated in Figure 2E). A subject-specific helmet was created by expanding the subject’s FreeSurfer reconstructed scalp surface by 2 mm. For co-registration, we performed digitization using a Fastrak Polhemus system (Polhemus Corp., Colchester, VT, USA). The head-to-MRI transform was estimated by aligning 3 digitized fiducial points (nasion, left and right pre-auricular points) with the same landmarks identified from the MRI. A device-to-head transformation was estimated by aligning 5 pre-determined reference points on the subject-specific helmet with the corresponding points in the 3D model of the helmet. A total of 16 sensors were placed in the helmet holders covering the contralateral somatosensory cortex.

The data were high-pass filtered at 4 Hz and low-pass filtered at 150 Hz to preserve the shape of early N20 components. Due to inherent noise in the OPM sensors, a low-pass filter was necessary. Background noise was removed using the signal space projection (SSP) method [29] in SQUID-MEG and using homogeneous field correction (HFC) in OPM-MEG [30]. All the analysis was performed using MNE-Python [31].

## 3. Results

### 3.1. Coil Designs

Figure 4 and Figure 5 show the stream functions, discretized current loops, the connected current paths from opmcoils, and the front and back copper layers of the two PCB halves in KiCad for each of the six coils ($B_{x}$, $B_{y}$, $B_{z}$, $G_{xz}$, $G_{yz}$, $G_{zz}$). We note that the $G_{xz}$, $G_{yz}$, $G_{zz}$ coils were similar to $B_{x}$, $B_{y}$, $B_{z}$, respectively; however, their winding patterns were simpler in comparison. This is because the $B_{x}$, $B_{y}$, $B_{z}$ coils needed to generate a sharp change in the field from a uniform value to zero at the edge of the target region while the $G_{xz}$, $G_{yz}$, $G_{zz}$ coils simply generated a gradually varying field. The six pairs of coils installed in our one-layer magnetically shielded room (Maxshield, Imedco) are shown in Figure 2E.

### 3.2. Theoretical vs. Empirical Resistance and Efficiency

The length of the conducting path, the theoretical and measured values of resistance with and without connected segments, and the efficiency of our coils with and without the mu-metal shield are shown in Table 1.

The length of the conducting path in opmcoils also considered the length of the connecting segments and their reverse paths and was up to 19 m longer (8% of the total length) than the length of the current loops only. The theoretical resistance agreed with the measured resistance though the measured resistance was systematically higher. The theoretical resistance considering the connecting segments did not fully account for this systematic difference. We hypothesize that the solder joints may be responsible for the discrepancy. However, comparable resistance values have been reported in previous studies [14], which successfully operated OPM nulling coils.

In terms of efficiency, the most efficient coils were the z-direction coils ($B_{z}$ and $G_{zz}$) as their optimized design approximates a Helmholtz design. The $B_{x}$ and $B_{y}$ coils had a similar theoretical efficiency (1.4 nT/mA). This was expected as the two coils are approximately rotated versions of each other. We noticed that the measured efficiency of the $B_{y}$ coil was slightly lower (1.3 nT/mA) than the expected theoretical efficiency (1.4 nT/mA). Our initial computation of the theoretical efficiency did not account for the effect of the mu-metal shield. We expected that the high permeability mu metal could impact the measured efficiency. Indeed, since the nulling coils were placed asymmetrically inside the shielded room (closer to the floor) along the y-axis, the distortion was largest in this direction. After considering the effect of shielding, the experimentally measured coil efficiency was within 6% of the theoretically measured coil efficiency for all the coils.

### 3.3. Remnant Field Mapping in Target Area

The spatial maps of the residual field in the x, y, and z directions after turning on the $B_{x}$, $B_{y}$, and $B_{z}$ nulling coils, respectively, are shown in Figure 6. By manually adjusting the applied current, we were able to reduce the background field to 2 nT. Based on the figure, we did not observe a spatially consistent gradient field that could be canceled with our gradient field-nulling coils. Therefore, we did not apply gradient field nulling for the remaining experiments. Since we operated the sensors in a lightly shielded one-layer room, the background field drifted in time, which could affect our estimate of the applied current. Thus, the final background field nulling could be further improved if closed-loop nulling were to be implemented.

### 3.4. Remnant Field Mapping in Helmet

**Figure 7:**
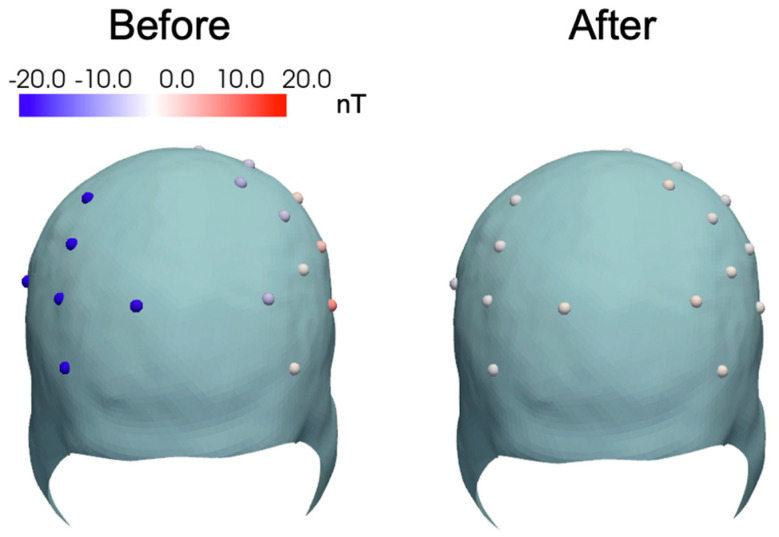
Map of residual magnetic field normal to the helmet surface, measured along the sensitive axis of the OPM sensor, with all the uniform field-nulling coils turned on. The maximum residual field in the OPM sensors after field nulling was 2 nT.

Figure 7 shows the spatial map of the residual field along the sensitive axis of the OPM sensor before and after turning on the uniform field-nulling coils, respectively. The maximum measured background field on the helmet surface was reduced from 21 nT to 2 nT, indicating that the field-nulling system could successfully remove the remnant field regardless of the OPM sensor orientation.

### 3.5. MEG Measurement and Analysis of Somatosensory Evoked Fields (SEF)

**Figure 8:**
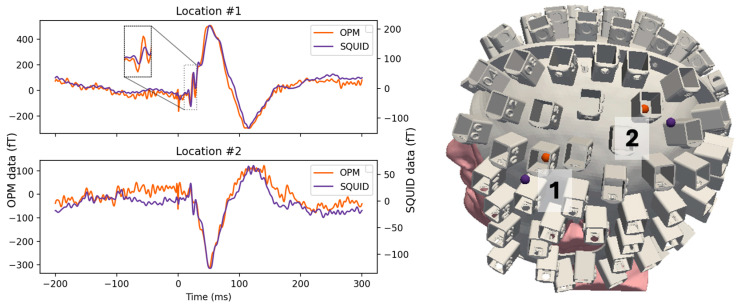
Averaged somatosensory evoked fields (OPM and SQUID) measured at two locations in response to median-nerve stimulation (**left**). A subject-specific helmet was created for the experiments by expanding the Freesurfer reconstructed scalp surface (**right**).

After confirming that we could remove the remnant field on the helmet surface, we used it for a median-nerve stimulation experiment using OPM-MEG while the field-nulling system was used in the one-layer shielded room. Averaged event-related fields (ERF) in two OPM sensors (N = 607) and two SQUID magnetometers (N = 496) from comparable sensor locations are shown in Figure 8. We note that the amplitude of the OPM data was about 2.5 times the SQUID data; however, the noise in the OPM was also proportionally higher. Nonetheless, the OPM measurement was remarkably similar to the SQUID data, showing major ERF components of the somatosensory evoked field (SEF): an early N20 response (see inset) followed by a P50 deflection and a later component peaking at about 100 ms.

### 3.6. Open-Source Software and Hardware

**Figure 9:**
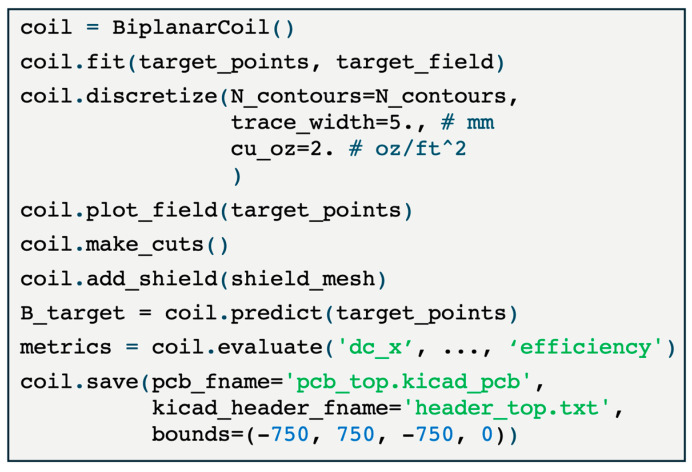
Application Programming Interface (API) for opmcoils enabling users to obtain biplanar nulling coils for PCB manufacturing.

Our study was made possible by the open-source software bfieldtools [25,26]. Bfieldtools is a general-purpose software for solving the coil design problem on arbitrary mesh surfaces. Using this software as our base, we implemented additional functionality in opmcoils for the practical issues encountered when manufacturing biplanar nulling coils. Our software, opmcoils (Figure 9), provides a template code to design biplanar nulling coils to null the uniform and gradient components of the background field. The optimized discrete current loops can be connected into a continuous path using an interactive tool and exported to and from KiCad for PCB design. A key feature of our software is that parameters can be provided in physically realizable units: the trace width of the PCB in mm, the thickness of the copper layer in oz, and the coil efficiency in nT/mA. Users can also easily compute the impact of the mu-metal shielded room on the coil efficiency [25,26]. In addition to the software, we also open-sourced the hardware components of our nulling coils. The Gerber files required to print the PCBs, the 3D models used for evaluating them, and the parts list to mount the PCB frame will all be available in our repository. Taken together, we provide users with the complete open-source toolkit necessary to download, adjust, and set up PCB-based nulling coils in their MEG centers.

## 4. Discussion

***Advantages of open-source PCB design:*** Manually wound nulling coils have been reported and successfully used in the OPM-MEG literature; however, they are labor-intensive due to various challenges in winding the wires. The wire lengths can be over 1000 m and the discrete current loops must be connected into a single continuous path without introducing stray fields. Along with the fact that the winding directions can be either clockwise or anticlockwise, this means that the physical realization of the coils is a complex process. Therefore, it is difficult to be precise, and errors are likely and not easily fixed because the wires are glued on a printed sheet of paper. Another method to construct the coils is to manually wind the coils in 3D-printed grooves [32]. While this technique can be beneficial when the stream function is constrained to a curved surface like an MRI coil, the spatial dimensions for the biplanar coil make 3D printing challenging. It also involves error-prone manual winding.

When we started the design of the nulling coils, easily manufacturable coil designs were not available. Furthermore, the technical implementation details to convert mathematical equations into physical realizations of coils were not easily available. We have streamlined the development process, and we share not only the code used in the development but also the coil designs as Gerber files. These Gerber files can be sent to a PCB manufacturer to be printed and used to null fields in any OPM-MEG center. Unlike manually wound nulling coils, PCBs are geometrically precise and easily replicable. Our study can be used to standardize new nulling coil systems for OPM-MEG systems in different environments and test their performance. Ultimately, we hope this will lower the barrier to entry in using OPM-MEG.

Another advantage of our open-source software-hardware PCB design is that the manufacture cost is lower. The open-source approach makes it possible to manufacture the coils at scale and reduces the costs for both vendors and users of OPM-MEG systems. Each of the coil pairs costs around $2000 and the current drivers cost $2400 each. Including the cost of installation, we estimate that our system costs less than $20,000 in total. We envision that a non-commercial nulling coil system will be significantly cheaper than commercial alternatives and will stimulate the MEG community to develop shared expertise and documentation for the use of these systems. Through further optimization of the designs, such as a balanced biplanar coil, the cost can be reduced even more [15].

***Design flexibility:*** In a PCB-based manufacturing system, the coils are chemically etched, the total amount of copper used remains the same, and therefore the cost remains constant regardless of the coil design. Due to this flexibility in design, the PCB manufacturing process can be used to produce more ambitious coil designs which target higher efficiency or multiple target areas. Indeed, the efficiency can be enhanced easily by increasing the number of discretized current loops and the resistance can be minimized by increasing the trace width. Even though high efficiency was not necessary in our shielded room, the design can be readily modified for use in other recording environments with challenging background fields.

The coils were divided into halves along the symmetry axis. For some coils with double symmetry, users may opt to print the coils in smaller segments, such as quarters. However, this approach introduces additional solder joints, creating potential failure points in the conducting path. Our design with the coil divided along the symmetry axis results in only two solder joints for the x and y coils, thus striking a balance between manufacturability and structural reliability.

***System installation:*** During installation, care must be taken to route the input wires so that stray fields are minimal. The shielded room can also distort the field and change the efficiency of the coils. The lower edge of our $B_{y}$ coil was 10 cm from the mu metal floor of our shielded room, which reduced the efficiency by 0.1 nT/mA. Raising the coil may improve the efficiency; however, the height of the coils was determined by practical considerations such as the height of the subject chair. One can redesign the coil accounting for the effect of the shielded room, but in our large shielded room, the measured efficiency was sufficient to operate the OPM-MEG system. To reduce the cost of OPM-MEG, compact and portable shielded rooms are being developed [33]. Implementing active shielding in such environments will certainly require incorporating the shielding effects into the coil optimization process. This can be achieved using an equivalent coupling matrix, defined as ${C_{B_{eq}}=C}_{B_{coil}}-C_{B_{shield}}C_{U_{shield}}^{-1}C_{U_{coil}}$ during the optimization process [25,26].

An important factor during installation is reducing mechanical vibrations while ensuring structural stability. To prevent the PCB from bending under its own weight, we reinforced it with an aluminum frame, and neoprene strips placed between the coils helped dampen vibrations. New laboratories interested in adopting our manufacturing approach can find a complete list of the materials required for assembly on our project website.

***Dynamic nulling vs*. *static nulling:*** In this study, we provide an open-source PCB-based field-nulling system to simplify the manufacturing process and enable reproducibility. We have demonstrated that this can be used to reduce the background field up to 2 nT. However, the background field shows significant temporal variation, particularly in lightly shielded environments. To enable optimal OPM performance in such environments, we recommend that laboratories use dynamic nulling that adjusts the current amplitude in real time.

***Recommendations for new laboratories:*** Before using our nulling coils, we recommend that users perform a simulation to test the efficiency and homogeneity of the fields. While the effect of shielding was minimal in our large room, it may be necessary to optimize for the effect of the shield if the distance between the shield walls and the coils is small. To avoid effects of higher-order field components, we recommend that users use a target region closer to the center of the room and away from any para- or ferromagnetic materials. Even though higher-order field components can be theoretically nulled, it may be difficult to characterize their spatial distribution. We recommend that new laboratories use an automatic current tuning procedure [16] in conjunction with the nulling coils shared on our project page.

## 5. Conclusions

This study is a demonstration of the use of PCB-based field-nulling systems for OPM-MEG. We successfully designed, developed, and tested the performance of our open-source PCB-based nulling coils for removing uniform background fields and selected spatial gradient components. The PCB design allowed us to obtain empirical efficiency matching the theoretical efficiency to within 6%. The remnant field mapping demonstrated that the background field can be reduced to less than 2 nT after using our nulling coils. We also conducted a median-nerve experiment and demonstrated that the signals obtained using SQUID-MEG and OPM-MEG were comparable when operated in conjunction with our nulling coils. Our open-source field-nulling system could lower the barrier to entry for the use of OPM-MEG in clinical and neuroscience applications. By leveraging the precision and replicability of PCBs, nulling coil systems can be standardized and evaluated in different environments. In summary, our study streamlines the development process, enables efficient manufacture, reduces costs and manual labor, and improves reproducibility.

## Figures and Tables

**Table 1:** Theoretical and measured properties of the nulling coils. Our geometrically accurate design enables a close match between the theoretical and measured values.

Coil	Length (m)		Resistance (Ω)			Efficiency (nT/mA or nT/m/mA)		
	Theoretical		Theoretical		Measured	Theoretical		Measured
	Bfieldtools	Opmcoils	Bfieldtools	Opmcoils		Without Shield	With Shield	
*B*_x_	217.4	236.5	10.7	11.6	18.2	1.5	1.4	1.4
*B*_y_	214.3	232.2	10.5	11.4	12.8	1.4	1.3	1.3
*B*_z_	164.5	170.6	8.1	8.4	12.5	6.4	7.1	7.1
*G*_xz_	159.3	164.9	7.8	8.1	11.8	7.5	7.8	7.9
*G*_yz_	155.8	163.2	7.7	8.0	11.8	7.8	7.9	8.4
*G*_zz_	110.1	114.6	5.4	5.6	7.7	15.4	16.4	15.4

## Data Availability

The software and the Gerber files associated with the nulling coils can be found here: https://opm-martinos.github.io/nulling_coils. The original data presented in the study are openly available at https://github.com/opm-martinos/opm_coils/tree/main/examples/data.

## Abbreviations

The following abbreviations are used in this manuscript:

OPM	Optically pumped magnetometer
MEG	Magnetoencephalography
PCB	Printed circuit board
SEF	Somatosensory evoked field
SQUID	Superconducting quantum interference device
ERF	Event-related field
CAPE	Cross-axis projection error
